# Neuronal oscillations reveal the processes underlying intentional compared to incidental learning in children and young adults

**DOI:** 10.1371/journal.pone.0182540

**Published:** 2017-08-02

**Authors:** Moritz Köster, André Haese, Daniela Czernochowski

**Affiliations:** 1 Center for Cognitive Science, Technical University Kaiserslautern, Kaiserslautern, Germany; 2 Faculty of Education and Psychology, Free University Berlin, Berlin, Germany; University of Akron, UNITED STATES

## Abstract

This EEG study investigated the neuronal processes during intentional compared to incidental learning in young adults and two groups of children aged 10 and 7 years. Theta (3–8 Hz) and alpha (10–16 Hz) neuronal oscillations were analyzed to compare encoding processes during an intentional and an incidental encoding task. In all three age groups, both encoding conditions were associated with an increase in event-related theta activity. Encoding-related alpha suppression increased with age. Memory performance was higher in the intentional compared to the incidental task in all age groups. Furthermore, intentional learning was associated with an improved encoding of perceptual features, which were relevant for the retrieval phase. Theta activity increased from incidental to intentional encoding. Specifically, frontal theta increased in all age groups, while parietal theta increased only in adults and older children. In younger children, parietal theta was similarly high in both encoding phases. While alpha suppression may reflect semantic processes during encoding, increased theta activity during intentional encoding may indicate perceptual binding processes, in accordance with the demands of the encoding task. Higher encoding-related alpha suppression in the older age groups, together with age differences in parietal theta activity during incidental learning in young children, is in line with recent theoretical accounts, emphasizing the role of perceptual processes in mnemonic processing in young children, whereas semantic encoding processes continue to mature throughout middle childhood.

## Introduction

Learning is a continuous process that accompanies cognitive processing and often takes place without the intention to memorize (incidental encoding, [1]). In other situations, the intentional acquisition of new information is emphasized in an effort to enhance the learning process, e.g., when children and young adults learn novel information in the context of formal education (intentional learning). Notably, during intentional learning, strategic processes can be employed to putatively enhance the learning outcome, for instance when attention is actively directed to relevant stimulus features. However, to the best of our knowledge, the neuronal oscillatory processes underlying intentional compared to incidental learning in children and adults have not been investigated so far.

From a developmental perspective, it is assumed that episodic memories do not only allow children to re-experience and share episodes from their past, but gradually change the structure of the maturing memory system [2, 3]. When children learn new details, with repeated presentations these details become decontextualized to form more abstract, conceptual representations [4, 5]. In turn, increasingly abstract representations allow children to process novel stimuli more efficiently and to integrate novel information into increasingly differentiated semantic networks. In the context of formal education, typically detailed factual knowledge (i.e. semantic information) needs to be retrieved independent of the context in which is was acquired (i.e. episodic information). Despite these qualitative changes in the way novel information is encoded in young as compared to older children and adults, and the profound implications for learning and retrieving information in an educational context, only a handful of studies have addressed maturational changes in memory encoding processes so far.

Using behavioral methods [6, 7], only the outcome of a learning process can be evaluated. Thus, to understand the mechanisms underlying the formation of new memory traces under different encoding conditions, it is necessary to study the brain activity at the time of encoding as a function of memory outcome. In subsequent memory paradigms the neuronal activity during encoding is compared between items which are remembered and items which are forgotten in a subsequent memory test. Functional neuroimaging (fMRI) studies with adults indicate that networks within the prefrontal cortex (PFC), the parietal cortex and the medio-temporal lobe (MTL) show higher activity for later remembered, as opposed to later forgotten items [8]. Notably, the PFC and the parietal cortex in particular have been shown to undergo structural changes with ongoing maturation [9]. Likewise, maturational changes are found in the MTL [9, 10] as well as for the connectivity between MTL and PFC structures [11]. Furthermore, research on oscillatory brain activity revealed new insights into the neuronal processes and mechanisms underlying successful memory formation [12–14]. Neuronal oscillations reflect the rhythmical interplay within and across cell assemblies and facilitate neuronal communication [15, 16] and memory processing [12]. Klimesch and colleagues [17, 18] were the first to demonstrate a close link between successful encoding and increased theta (3–8 Hz) as well as decreased alpha activity (10–16 Hz) in the EEG of adults. Later scalp and intracranial EEG studies replicated and extended these initial findings: Successful encoding was consistently associated with increased theta and decreased alpha activity in incidental [19–21] and intentional encoding tasks [22, 23]. For instance, Friese et al. [19] found increased prefrontal theta power for later remembered as opposed to later forgotten stimuli, accompanied by decreased alpha activity in widespread frontal and parietal cortical networks for later remembered items. However, despite these similarities, former studies indicate that alpha and theta activity vary with specific demands of the encoding task, suggesting that these frequencies dissociate between different processes involved in encoding [13]. Theta oscillations are assumed to reflect binding and sequential ordering processes underlying successful encoding [14, 24, 25]. In the human EEG, theta oscillations during mnemonic processing are furthermore thought to indicate the interplay between cortical processes and hippocampus-dependent binding mechanisms [25–27], which form the basis for relational memory processes in the hippocampus [28]. On the other hand, alpha activity, specifically in the upper alpha range (> 10 Hz), has been shown to decrease with the semantic demands of the encoding task in previous subsequent memory studies [18, 20, 21], suggesting that upper alpha activity is a marker of semantic encoding processes [29]. Presumably, alpha suppression facilitates semantic processing by gating the communication between task-relevant brain regions [30]. A study by Hanslmayr and colleagues [20] specifically tested the functional dissociation between theta and alpha oscillations during encoding contrasting different encoding conditions: When participants were asked to order letters in a word (a task requiring perceptual elaboration and sequencing), successful encoding was associated with a selective increase in theta activity in frontal and parietal networks. Conversely, during an animacy decision (a task requiring semantic processing), successful encoding was reflected in decreased alpha power in frontal regions. Hence, modulations in the theta- and alpha-band during the formation of novel memories are assumed to indicate perceptual processes like binding and ordering, and semantic processes, respectively. Despite the accumulating evidence in adults, neuronal oscillations and the neuronal and cognitive mechanisms underlying memory formation have not been investigated throughout middle childhood so far [31, 32].

Empirical support for the notion that the mechanisms underlying encoding processes might change along with development comes from behavioral studies [33] and from two recent neuroimaging studies that report age differences in the brain networks active during memory encoding: Ofen and colleagues [34] found increased activity in PFC and MTL regions for later remembered as compared to later forgotten items in both children and adults. However, higher memory performance was related to increased activity in specific PFC regions in adults, but not in children. This age difference in task-related activity is in line with the notion that additional processes support memory formation in adults compared to children, for instance an increased reliance on meta-memory and strategic processes with ongoing development [35]. In a second study, Maril and colleagues [36], using a semantic encoding task, reported a stronger engagement of cortical regions associated with semantic processing in adults, whereas children engaged networks related to perceptual processing. Based on these findings, Ofen and Shing [2] proposed a qualitative shift from a perceptual to a semantic focus in the memory system during childhood. Furthermore, although former studies contrasted intentional and incidental encoding with respect to functional brain networks [37] and event-related potentials [38], to the best of our knowledge, differences in neuronal oscillatory activity between intentional and incidental encoding where not investigated thus far.

**Fig 1:**
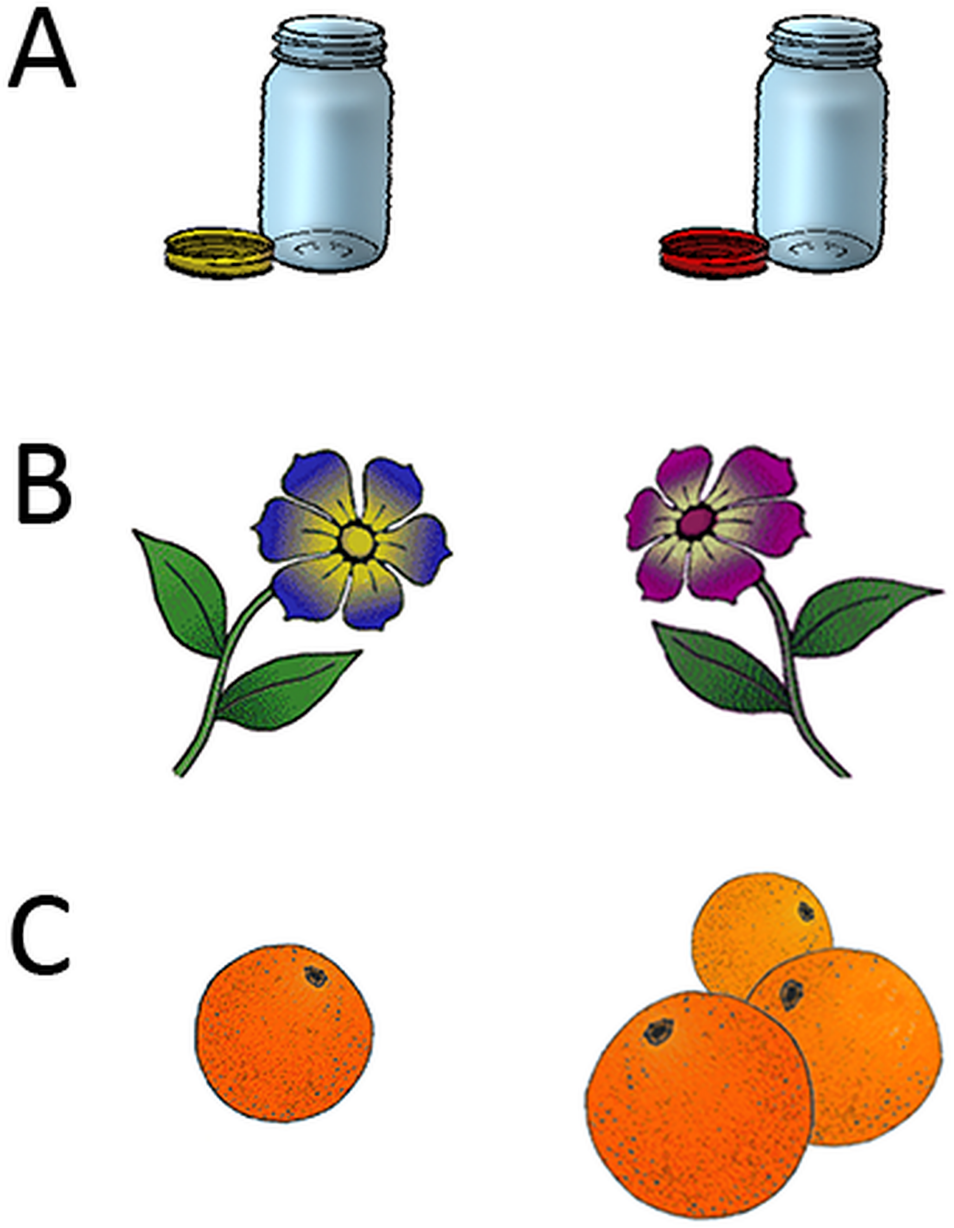
Sample stimuli used in the memory task. During retrieval, participants were asked to indicate whether or not perceptual features of the objects were changed, relative to the first presentation during encoding, as illustrated for three object pairs (A: color; B: orientation and color; C: quantity and orientation). Note that not necessarily only one dimension was changed between encoding and test.

In the present study, we used alpha- and theta-band oscillations in the EEG to investigate the processes underlying intentional encoding in 7-year-olds, 10-year-olds and young adults, when compared to an incidental encoding condition. We used a semantic encoding task with pictorial stimuli (see Fig 1) and tested memory for the stimuli and their perceptual features during subsequent retrieval. This is, participants were asked to indicate whether or not perceptual features of the items were changed relative to the encoding phase. Based on a recent theoretical account by Ofen and Shing [2], we expected that in young children the overall activity during encoding would predominantly rely on perceptual binding processes, whereas semantic processes should gradually complement these processes in older children and adults. Accordingly, we predicted fewer developmental changes for theta-band activity, being associated with perceptual binding, but higher alpha suppression with increasing age, indicating the maturation of semantic processes. The main goal of this study was to contrast intentional and incidental encoding to assess how encoding operations are modulated when participants intentionally learn the stimulus material for the subsequent memory test. During intentional encoding, we predicted higher levels of theta power as an indicator for the elaboration of perceptual features, which were relevant for subsequent retrieval in the present paradigm. Furthermore, we explored differences between intentional and incidental encoding operations with ongoing development. Adults, and possibly older children, were expected to recruit strategic processes during intentional encoding.

## Materials and methods

### Participants

Participants were young adults (first-year undergraduates, *n* = 18, *M*_age_ = 21;6 years, *Range*_age_ 20 – 23), older children (attending fifth grade, *n* = 19, *M*_age_ = 10;6 years, *Range*_age_ 9;7 – 11;4), and young children (attending second grade, *n* = 19, *M*_age_ = 7;8 years, *Range*_age_ 7;4–8;1). All participants had normal or corrected-to-normal visual acuity and were free of neurological or psychiatric disorders according to self-report. After the procedure and the EEG technique were explained, all participants agreed to participate in a written form. While students signed a standard consent form, children signed an assent form stating the purpose and methods of the research in a child-friendly language, with a special emphasis on a voluntary participation that could be withdrawn at any point in time. This form was read aloud to the children while their parents were present. In addition, their parents signed a more detailed consent form, e.g. with additional information about the methods employed and confidentiality procedures.

All participants, as well as children's parents were given the opportunity to ask any question before agreeing to participate in this study. The study conformed to the Code of Ethics of the American Psychological Association and was approved by the ethics committee of the University of Düsseldorf. In total, eleven additional participants were not included in the analysis due to low behavioral performance, i.e. two standard deviations below group average (adults: *n* = 1; older children: *n* = 3; younger children: *n* = 2), excessive artifacts (adults: *n* = 2), incomplete or insufficient data (younger children: *n* = 3), or self-report of less than four hours of sleep in the night before the experiment (adults: *n* = 1).

### Stimuli and procedure

The stimulus set consisted of 200 colored drawings of objects, taken from a picture set created by Rossion and Pourtois [39]. Objects were familiar to children in the investigated age groups (e.g. animals, plants). In addition, we created slightly changed exemplars for 60 of these images by editing perceptual features, such as size, orientation, quantity and color of the objects to assess memory for specific object features (see Fig 1). All participants performed an incidental encoding task, and were not aware that their memory would subsequently be assessed. During incidental encoding, 80 objects were presented in a randomized order while participants made a semantic judgment (indoor vs. outdoor decision). After two minutes, participants completed an unannounced retrieval task with 120 pictures: 40 pictures were the same exemplars as shown during the encoding phase, whereas 40 pictures were changed exemplars and 40 new pictures served as distracters. During retrieval, participants were asked to indicate whether a picture was presented as previously shown during encoding (identical), as a changed exemplar (changed) or not shown before (new). After a short break, participants performed a second, intentional encoding block: This time, they were asked to memorize the presented pictures while still performing the indoor vs. outdoor decision. Note that participants were now also familiar with the demands of the retrieval task (i.e. to distinguish between changed and identical item repetitions and distractor items). During intentional encoding, 40 novel pictures were studied along with 40 items re-learned from the previous phase, which were not further analyzed. Finally, memory for these items was assessed in a second retrieval phase. Note that incidental and intentional encoding can only be assessed in this order, because once participants are instructed to learn, they would likely expect a subsequent memory test irrespective of the explicit task instructions. During encoding, each stimulus was presented for 1 s, followed by the response options (indoor/outdoor), presented on the screen until a response was given. During retrieval, target stimuli were presented along with the response options (i.e. identical/changed/new), which remained on the screen until a response was given. Each trial was preceded by a blank screen (1 s) and a fixation cross (1 s). The procedure was demonstrated in 8 training trials prior to the encoding task, and 12 training trials prior to the retrieval task (with 4 identical, 4 changed and 4 distractor items).

First, to identify age-related changes in neuronal activity associated with the processing of the stimulus material during encoding, we analyzed all items with correct subsequent feature judgments (as identical or changed) of both phases (incidental and intentional). Second, to assess the neuronal correlates of intentional encoding, we contrasted successfully encoded trials between the intentional and incidental encoding conditions. In memory tasks, adults often outperform children [40, 41]. Hence, typically fewer correct trials are available for assessing neuronal correlates of memory in children compared to adults. Therefore, we opted for a moderate list length and low interference between response options to avoid a systematically lower number of correctly remembered trials for children compared to adults. Consequently, the present design results in an overall high memory performance across age groups, and a low number of items that were later forgotten or not correctly classified as identical or changed, which could thus not be analyzed. Thus, the approach of the present study allowed us to better differentiate between intentional and incidental encoding, the main contrast of interest, while we were unable to contrast successful with unsuccessful memory encoding. The analyses of the neuronal activity during the retrieval phase are reported in a companion papers, one focusing on young adults [42] and one focusing on children [43].

### Behavioral data

Overall memory performance was analyzed by the means of corrected recognition scores (Pr; [44]), i.e., by subtracting the proportion of false alarms (i.e., new items misclassified as old) from the proportion of hits (i.e., correctly identified old items). Pr values were calculated irrespective of the correctness of feature judgments (i.e., as identical or changed), separately for intentional and incidental encoding. In addition, we compared the accuracy of feature judgments, i.e., the proportion of correct feature judgments relative to the total number of remembered items. Pr and feature accuracy scores were entered into mixed model ANOVAs with the factor Age Group (young children, older children, young adults) and the repeated factor Condition (intentional encoding, incidental encoding). For this and all subsequent analyses, Greenhouse-Geisser corrected *p*-values are reported along with uncorrected degrees of freedom. Effect sizes are quantified by partial eta squared η_*p*_^*2*^. Behavioral data were analyzed for all 56 subjects included in the EEG analysis.

### Electrophysiological recordings

**Fig 2:**
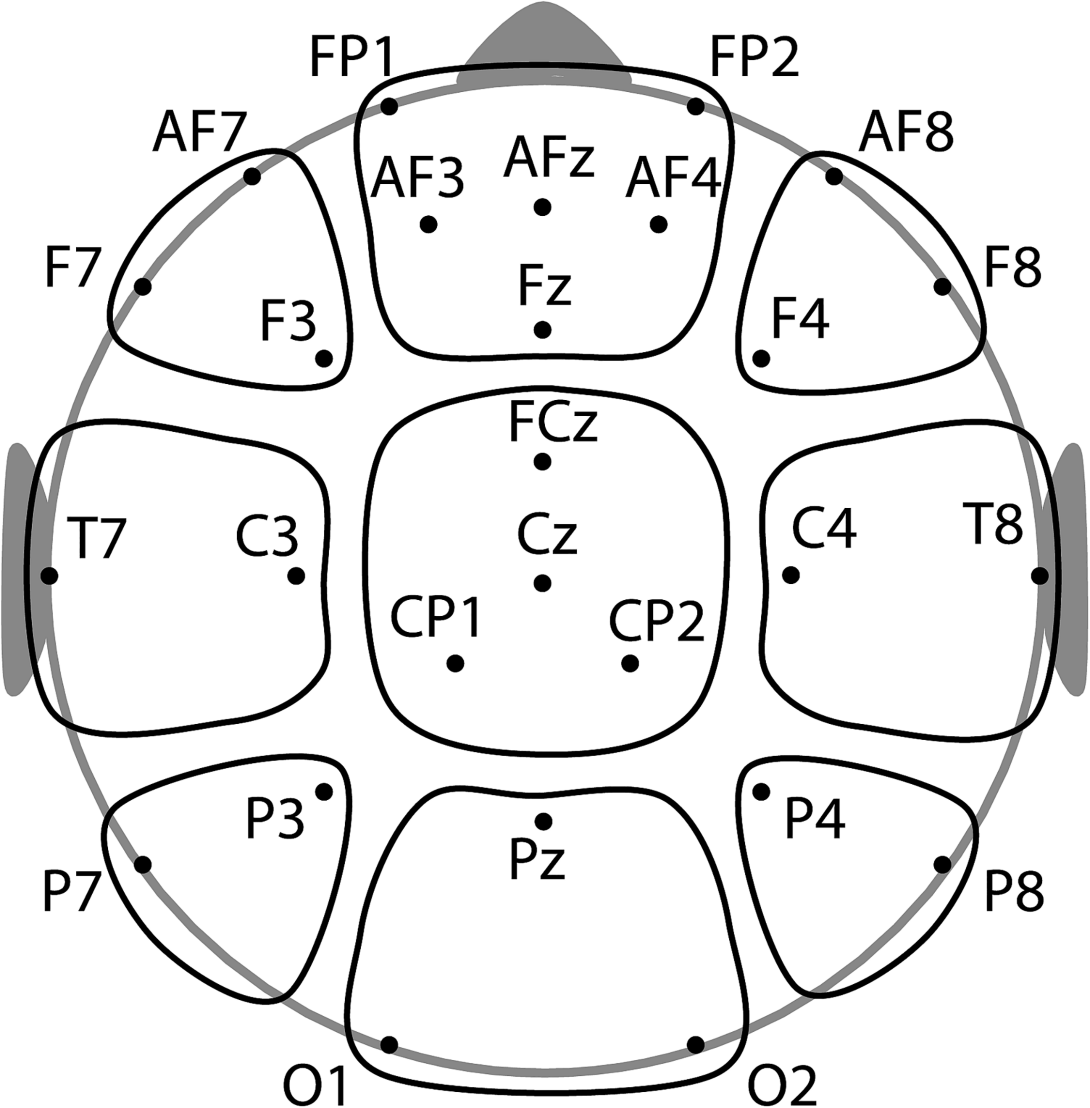
Electrode configuration and the clusters used for the statistical analyses. Electrodes were grouped along two dimensions, divided into 3 laterality (left, medial, right) by 3 caudality (frontal, central, parietal) clusters.

EEG was recorded from 27 active Ag-AgCl electrodes (see Fig 2) at a sampling rate of 500 Hz (EEG amplifier: BrainAmp S/N AMP 14092302DC). Frontal electrodes are over-represented in this electrode configuration to achieve a more fine-grained measure for frontal neuronal processes, i.e. frontal theta- and alpha-band oscillations. A horizontal and a vertical electroocculogram (EOG) were assessed to monitor eye movements and blinks. Electrode impedance was kept below 25 kΩ. Electrode FCz served as an online reference and was restored after offline re-referencing to the average reference, using the 19 electrodes of the standard 10–20 configuration. In line with prior EEG studies analyzing neuronal oscillations during mnemonic processing [19, 20, 27], we used an average reference for ease of comparison. To avoid a relative bias of frontal activity, only electrodes corresponding to the standard 10–20 system were included in the average reference (because frontal electrodes are over-represented in the electrode setup). EEG data were bandpass-filtered offline from 0.5 to 25 Hz. Prior to the analysis, up to three channels with remaining artifacts per subject were detected by visual inspection and interpolated. Eye blinks and horizontal eye movements were detected using an independent component procedure incorporated in the BrainVisionAnalyzer 2.0 software and removed after visual identification. Data were segmented into epochs of -500 to 2500 ms with respect to stimulus onset. Trials with muscular or technical artifacts were identified by predefined thresholds of signal change for each electrode within 200 ms intervals (100 μV for adults, 150 μV for older and 200 μV signal change for younger children) and removed. Participants with a minimal number of six trails in each condition remained in the analysis. However, in a secondary analysis we tested whether the results of the behavioral and EEG analyses would change when subjects with less then 10 trials in each condition (adults: n = 1, older children: n = 2, younger children: n = 5) were removed from the analysis. Importantly, all results reported in the present study remained statistically reliable when removing these participants from the analysis.

### Spectral changes in electrode space

Spectral amplitudes of oscillatory activity in the time-frequency domain were calculated by the means of complex Morlet´s wavelet transform, with wavelets of about 7 cycles between 0.5 and 20 Hz, in 0.5 Hz steps. Spectral changes were averaged over the trials of each subject, separately for each condition. Using this method, oscillatory signals are composed of both evoked oscillations, i.e. locked to stimulus onset, and induced oscillations, i.e. with a slight temporal jitter across trials [45]. Spectral amplitudes in the analyzed frequency ranges differed markedly between age groups, with higher signals in children as compared to adults. This is consistent with generally larger EEG signals in children in this age range. Therefore, analyses were based on relative signal changes upon stimulus onset, namely the relative increase or decrease in oscillatory activity in percent: Spectral amplitudes of each trial were first divided by a -300 to -100 ms baseline and then the baseline level was subtracted to set the baseline level to zero. By choosing a baseline ending prior to stimulus onset we avoided trial-related activity in the baseline, which might occur due to smoothing effects of the wavelet analysis [46]. Time-frequency plots were used to depict the relative signal changes during successfully encoded trials with correct subsequent feature judgments: Spectral amplitudes were collapsed across all electrodes and trials of both encoding conditions (intentional and incidental), separately for each age group.

The present analyses focused on stimulus-triggered changes in theta (3–8 Hz) and alpha (10–16 Hz) oscillations. As the theta- and the alpha-band vary largely between individuals [18], these frequencies were adjusted for each subject, based on all trials and both learning conditions, in a time window from 0 to 1000 ms: According to the properties of theta and alpha activity, we selected the frequency band with the highest increase between 3 and 8 Hz at frontal and posterior electrode sites as individual theta and the highest decrease between 10 and 16 Hz at as individual alpha. On average, individual theta frequencies were 5.3 Hz (*SD* = 1.7) for young adults, 5.7 Hz (*SD* = 1.3) for older children and 5.5 Hz (*SD* = 1.3) for young children; individual alpha frequencies were 13.8 Hz (*SD* = 1.9) in young adults, 12.6 Hz (*SD* = 1.7) in older children and 12.6 Hz (*SD* = 2.0) in younger children. Individual frequencies did not differ between the age groups for theta, *F*(2, 53) = 0.78, *p* = .691, and differed marginally in the alpha band, *F*(2, 53) = 2.79, *p* = .070. Topographical maps were used to illustrate the cortical distributions of changes in individual frequency bands associated with stimulus processing during encoding. Furthermore, we computed the corresponding difference between intentional and incidental encoding.

**Fig 3:**
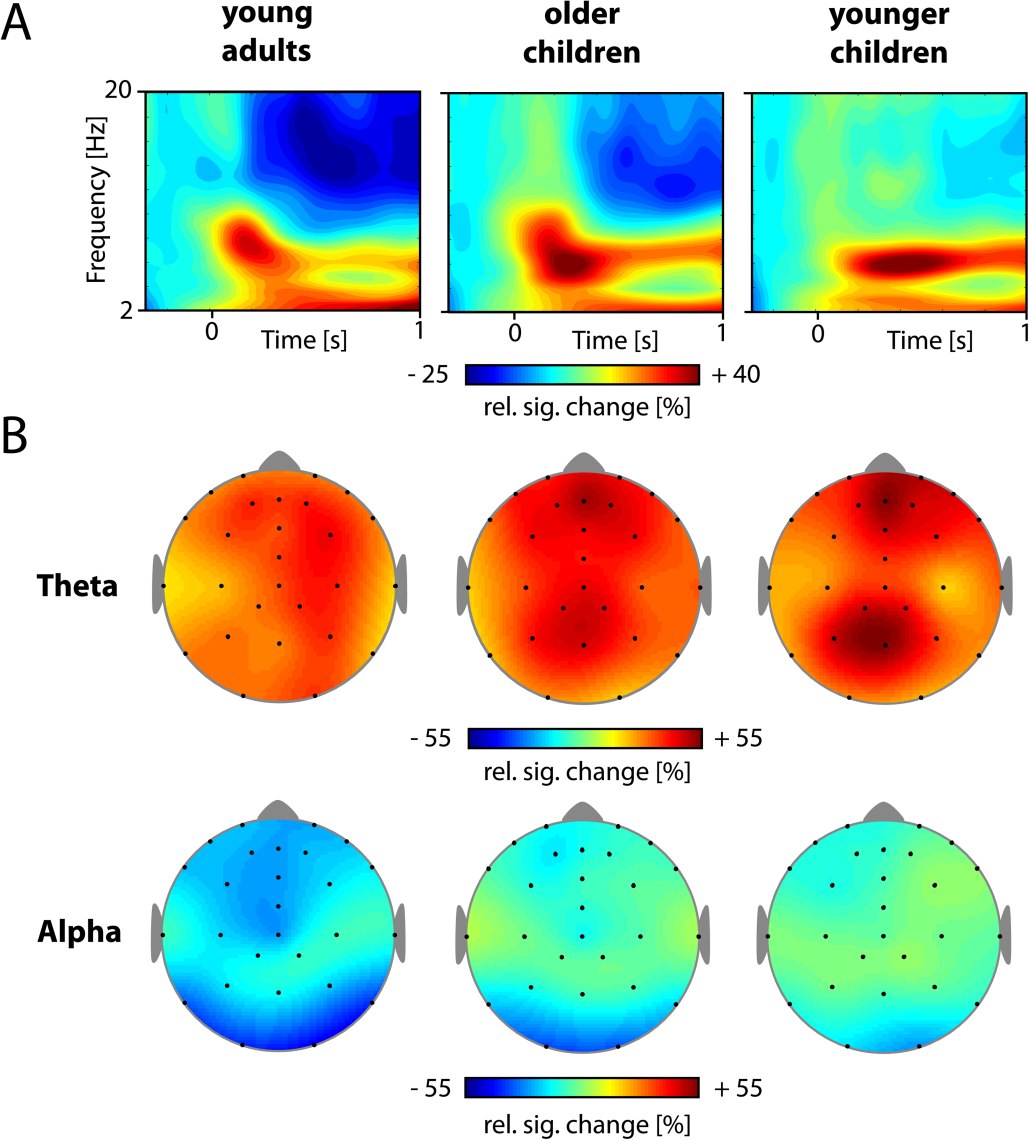
Spectral changes in theta and alpha activity during encoding. (A) Time-frequency plots illustrate the relative signal changes upon stimulus onset for successfully encoded stimuli (i.e., subsequently remembered items with correct feature judgments). The signal changes depicted here are averaged over all electrodes, all participants in each group and both learning phases (incidental and intentional). (B) Topographical maps for event-related changes in individual theta and alpha activity during encoding. Signal changes are averaged over the entire stimulus presentation (1s), all participants in each age group and both learning phases (incidental and intentional).

Because this is the first study to investigate neuronal oscillations during encoding in children, in a first step, we clustered all 27 electrodes into a 3 x 3 grid, according to their caudality and laterality (see Fig 2) for an overall statistical comparison between brain regions, age groups and conditions. Relative signal changes were averaged over the electrodes of each cluster and the time window of interest (0–1000 ms, corresponding to stimulus presentation), separately for each participant and condition. For both alpha and theta frequencies, mean signal changes were entered as dependent variables into mixed model ANOVAs with Caudality (frontal, central, posterior), Laterality (left, medial, right) and Condition (incidental, intentional) as repeated factors and Group (adults, older and younger children) as between-subject factor. In a second step, to further scrutinize the differences between incidental and intentional encoding, we focused on frontal and posterior electrode clusters. This was due to the close correspondence of grand mean topographies of theta and alpha activation across age groups (see Fig 3) and to the frontal and posterior alpha and theta networks identified in previous assessments with students [19, 27, 47]. Specifically, we entered the average signals over the electrodes of all frontal and posterior clusters and entered these into a Cluster (frontal, posterior), Condition (incidental, intentional) and Age Group (adults, older and younger children) ANOVA. Please note the labeling used for the factor of the ANOVA conducted in the first step (Caudality and Laterality) and the second step (Cluster), used consistently throughout this manuscript. Subsidiary ANOVAs and post hoc t-tests were calculated to follow up on significant interactions.

## Results

### Memory performance

Overall memory performance (i.e., old-new discrimination as indicated by Pr values) was comparable across age groups, *F*(2, 53) = 1.47, *p* = .239, η_p_^2^ = .05, and encoding conditions, *F*(1, 53) = 0.63, *p* = .432, η_*p*_^*2*^ = .01, see also Table 1, with no Age Group x Condition interaction, *F*(2, 53) = 0.44, *p* = .647, η_*p*_^*2*^ = .02. However, as expected, the accuracy of feature judgements increased in the intentional encoding phase, *F*(1, 53) = 12.38, *p* = .001, η_*p*_^*2*^ = .19. No difference in the accuracy of feature judgements was found between age groups, *F*(2, 53) = 0.70, *p* = .500, η_*p*_^*2*^ = .03, and the intentional learning instruction improved the accuracy of feature judgements in all age groups equally, Group x Condition interaction, *F*(2, 53) = 1.28, *p* = .286, η_*p*_^*2*^ = .05. Thus, importantly, age differences in the following EEG analyses cannot be attributed to age differences in memory performance.

**Table 1:** Memory performance.

	Young Adults	Older Children	Young Children
Old/new discrimination (Pr)			
Learning condition 1 (incidental)	.74 (.11)	.75 (.10)	.70 (.14)
Learning Condition 2 (intentional)	.75 (.10)	.73 (.14)	.68 (.16)
Feature Recognition (Ratio)			
Learning condition 1 (incidental)	.77 (.08)	.80 (.08)	.77 (.08)
Learning Condition 2 (intentional)	.84 (.08)	.83 (.06)	.80 (.12)

*Note*. Old/new discrimination and feature recognition accuracy (means and standard deviations) for the three age groups.

### Age differences in alpha and theta activity during encoding

To investigate age-related changes in neuronal activity during encoding, in a first step we compared age effects irrespective of learning conditions; Intentional and incidental learning phases were contrasted in further analyses as detailed below. Because this is the first study to investigate oscillatory activity during intentional and incidental encoding in children and adults, initially all electrodes were entered into an analysis of variance (ANOVA), clustered according to their laterality and caudality (Fig 2).

Time-frequency analysis of the changes in oscillatory brain activity during encoding revealed very similar signal changes upon stimulus onset in the three age groups (see Fig 3A), namely an increase in theta (3–8 Hz) and a decrease in alpha activity (10–16 Hz). These effects (theta power increase, alpha power decrease) were found in both phases for all three age groups and conditions (all |*t|*s > 2.32, all *p*s < .03; mean activity across all clusters, 0-1s, tested against zero). The topographies of changes in the theta and alpha band are displayed in Fig 3B.

Theta power was highest at medio-frontal and medio-parietal electrodes indicated by main effects of Caudality, *F*(2, 106) = 4.700, *p* = .014, η_*p*_^*2*^ = .08, Laterality, *F*(2, 106) = 6.794, *p* = .002, η_*p*_^*2*^ = .11, and the Caudality x Laterality interaction, *F*(4, 212) = 2.757, *p* = .046, η_*p*_^*2*^ = .05. There was no effect of Age Group, *F*(2, 53) = 0.523, *p* = .596, η_*p*_^*2*^ = .02, and no interactions of Age Group with the cluster dimensions Caudality and Laterality, all *F*s < 1.461, all *p*s > .232.

**Fig 4:**
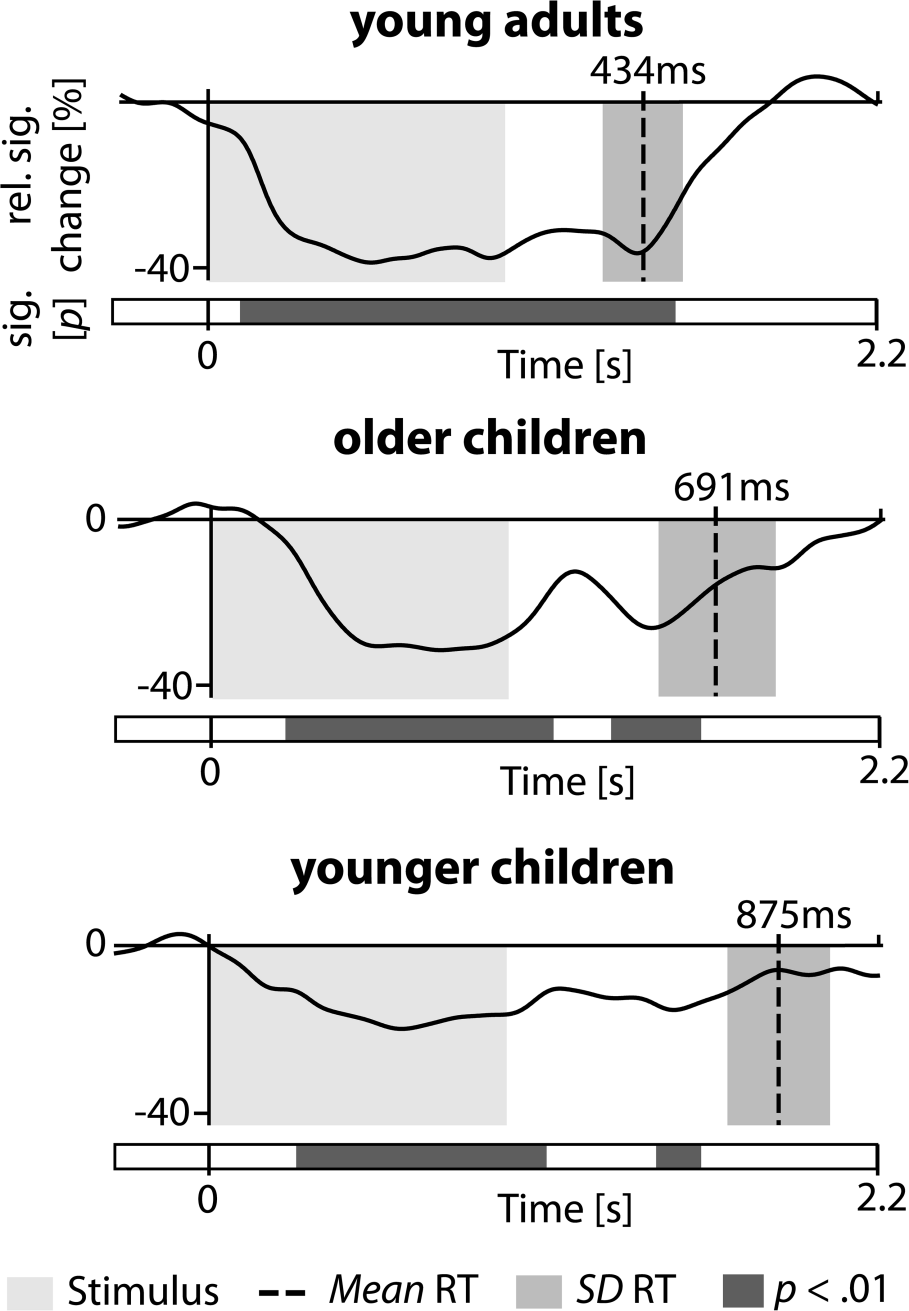
Posterior alpha suppression and corresponding response times. Graphs display the level of alpha-band suppression during and after stimulus presentation (stimulus: 0-1s, light gray), across electrodes of frontal and posterior electrode clusters and both phases. Dotted lines indicate mean response times for subsequent feature hits during encoding (±1 *SD*, gray), after stimulus offset until responses were given. Gray bars illustrate the result of a time-point-wise comparison against zero (*p* < .01).

Alpha suppression increased with age, *F*(2, 53) = 7.410, *p* < .001,η_*p*_^*2*^ = .22. In the topographical distribution of alpha power, we found main effects of Caudality, *F*(2, 106) = 46.171, *p* < .001, η_*p*_^*2*^ = .47, and Laterality, *F*(4, 106) = 22.786, *p* < .001, η_*p*_^*2*^ = .30. We also observed a Caudality x Laterality interaction, *F*(4, 212) = 5.489, *p* < .001, η_*p*_^*2*^ = .09, with higher alpha suppression over frontal and posterior cortical regions (see Fig 3A). This alpha reduction over frontal and posterior regions tended to increase with age, Caudality x Laterality x Age Group interaction, *F*(8, 212) = 1.874, *p =* .079, *η*_*p*_^*2*^ = .07. Focusing on frontal and posterior electrode clusters, confirmed the changes in frontal and posterior clusters with age, *F*(2, 53) = 8.176, *p* < .001,η_*p*_^*2*^ = .24. There was no effect of Condition nor any interaction of Condition with Cluster or Age Group, all *F*s < 1.740, all *p*s > .185. Thus, for a temporal analysis, we averaged the activity of frontal and posterior electrodes and both phases (see Fig 4). Alpha suppression was most pronounced in adults and returned to baseline level rather abruptly, around 500 ms after stimulus offset; it was less pronounced in older children and even lower in young children, but remained below baseline for prolonged time intervals in the younger age groups. As illustrated in Fig 4, these differences in the duration of alpha suppression closely correspond to age differences in response times during the semantic encoding task (for later remembered items only), *F*(2, 52) = 24.11, *p* < .001, *η*^*2*^ = .48, with longer response times for young children (*M*_*RT*_ = 875 ms, *SD*_*RT*_ = 190 ms) as compared to older children (*M*_*RT*_ = 691 ms, *SD*_*RT*_ = 233 ms), and longer response times for older children as compared to young adults (*M*_*RT*_ = 434 ms, *SD*_*RT*_ = 129 ms), all *t*s > 2.67, all *p*s < .011.

### Theta and alpha differences between intentional and incidental learning

**Fig 5:**
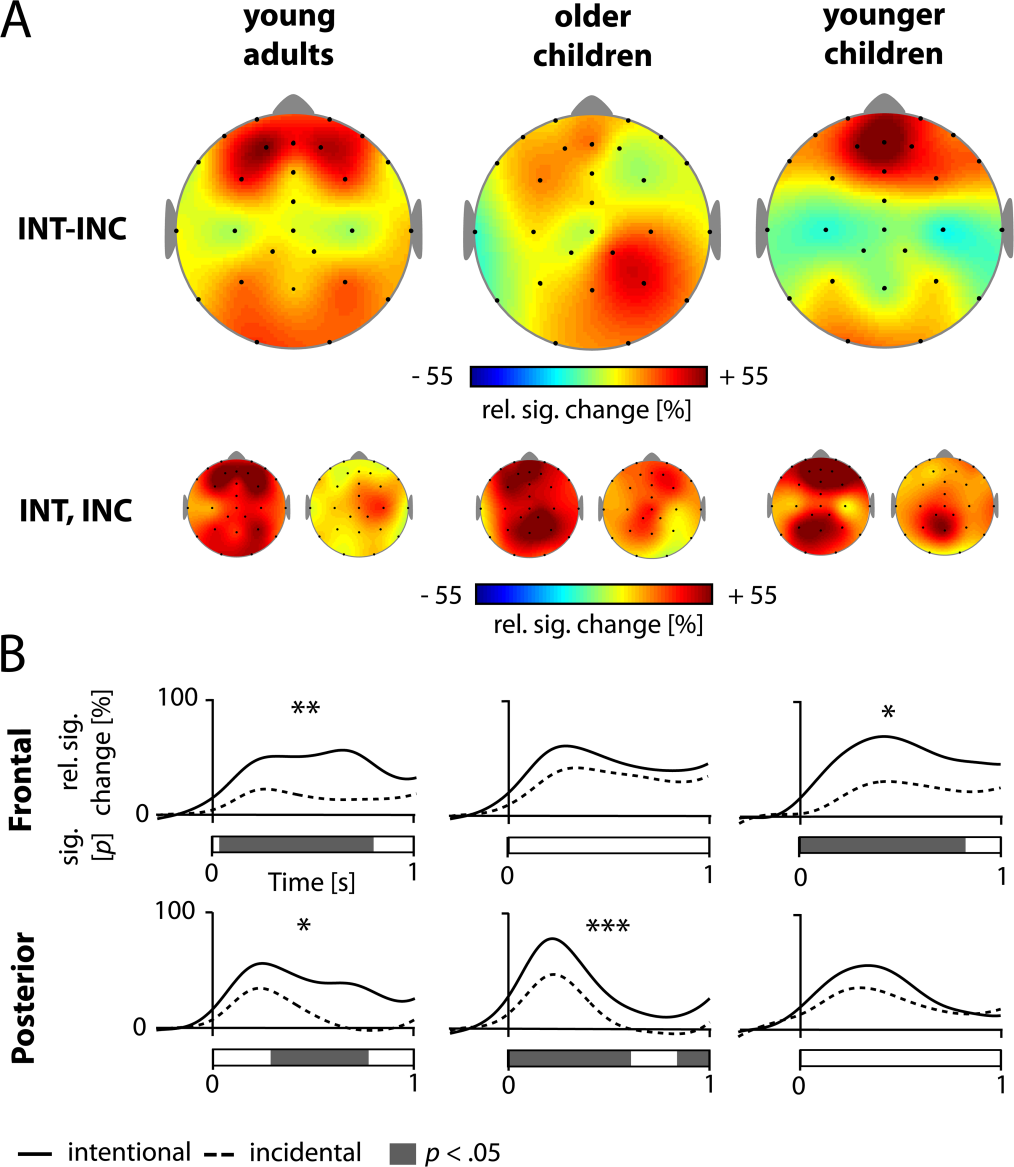
Intentional learning is reflected in the theta frequency. (A) Difference topographies of event-related changes in theta between intentional (INT) and incidental (INC) encoding, for the entire duration of stimulus presentation. (B) Relative signal changes (rel. sig. change), in percent, for intentional and incidental encoding, at all electrodes of frontal (upper panel) and posterior clusters (lower panel). Asterisks indicate the results of *post hoc* t-tests between encoding conditions, for the entire time window of stimulus presentation (*** *p* < 001, ** *p* < 01, * *p* < 05). Bars below the graphs illustrate the time course of these differences (*p* < .05, time-point-wise).

To assess the processes underlying intentional encoding, neuronal activity was contrasted between intentional and incidental learning conditions. Difference topographies for theta activity are displayed in Fig 5A, upper panel. Higher theta power was observed during intentional versus incidental encoding across age groups, as reflected in a main effect Condition, *F*(1, 53) = 16.668, *p* < .001, *η*_*p*_^*2*^ = .24. The difference in theta activity between intentional and incidental encoding was most pronounced at frontal and parietal electrodes, indicated by a Caudality x Condition interaction, *F*(2, 106) = 6.783, *p* = .005, η_*p*_^*2*^ = .27. Furthermore, it was somewhat less centered over the midline in the intentional phase as compared to the incidental phase, revealed by the interaction Laterality x Condition, *F*(2, 106) = 5.494, *p* = .005, η_*p*_^*2*^ = .09, as illustrated in Fig 5A, lower panels. Focusing on theta activity in frontal and posterior networks, we found an interaction between Cluster, Condition, and Age Group, *F*(2, 53) = 3.971, *p* = .025, *η*_*p*_^*2*^ = .13, as well as a trend for a Cluster x Group interaction, *F*(2, 53) = 2.419, *p* = .099, *η*_*p*_^*2*^ = .08. As visible in the lower panels of Fig 5A, in older age groups, parietal and frontal theta was pronounced in the intentional compared to the incidental condition. The youngest age group showed an increase in frontal theta activity during intentional encoding and similarly high levels of parietal theta activity during incidental and intentional encoding. Subsidiary Cluster x Condition ANOVAs, calculated separately for each group, confirmed this observation: The effect size of the Cluster x Condition interaction was much higher in young children, η_p_^*2*^ = .31, compared to older children, η_p_^*2*^ = .04, and adults, η_p_^*2*^ = .01, with *F*(1, 18) = 8.249, *p* = .010, *F*(1, 18) = 0.736, *p* = .402, and *F*(1, 17) = 0.131, *p* = .722, respectively. To follow up on this Cluster x Condition x Age Group interaction, we compared the theta activity during stimulus presentation for the frontal and posterior clusters between conditions. Specifically, statistical differences between conditions were assessed for each cluster and age group in post hoc t-tests (averaged over the entire 1 s time window). The time course of these differences is further described by the means of a sample point-wise paired *t*-test, see Fig 5B. Theta was significantly higher during intentional as compared to incidental encoding at frontal electrodes in the youngest and the oldest age group. In accordance with the significant Caudality x Condition interaction in the youngest group, time courses revealed differences between both encoding tasks in parietal theta activity for older children and adults, but not for the youngest group.

Relative signal changes in the alpha-band did not vary between conditions in the overall ANOVA, *F*(1, 53) = 1.456, *p* = .233, η_*p*_^*2*^ = .03, or the ANOVA focussing on frontal and posterior clusters, *F*(1, 53) = 0.745, *p* = .392, η_*p*_^*2*^ = .01. There were also no interactions of Condition with other factors in both analyses.

## Discussion

The present study examined developmental changes in intentional and incidental episodic memory encoding, to investigate memory processes enhanced during intentional encoding. Based on the theoretical model introduced by Ofen and Shing [2], we expected young children to rely predominantly on perceptual processing, and a gradual increase in complementing semantic processing for older children and adults. Encoding related activity across the intentional and the incidental encoding phase was associated with an increase in theta and a decrease in alpha activity across age groups. Alpha suppression over frontal and posterior cortical regions increased markedly with age. Importantly, intentional compared to incidental encoding was associated with higher theta activity, specifically over frontal and posterior cortical regions. Notably, in young children parietal theta activity was similarly high during intentional and incidental encoding, whereas it increased significantly for intentional compared to incidental encoding in young adults and older children. As outlined in the following paragraphs, these results are in line with the theoretical assumption that semantic encoding increases during childhood, whereas perceptual processes play a particular role during encoding in young children [2].

### Developmental shift from a perceptual to a semantic focus during encoding

Theta activity during encoding showed an overall increase across age groups. Importantly, theta activity increased during intentional as compared to incidental encoding, suggesting that intentional learning was associated with increased elaboration of perceptual features. More specifically, the formation of novel episodic memories depends on hippocampus-dependent binding processes [28]. The cortical theta rhythm is assumed to be a marker of the communication between neocortical and MTL regions [14, 25, 26] and, thus, to reflect the key mechanism underlying the ordering and binding of novel perceptual details, represented in gamma oscillations [14, 19, 27]. However, the detection of gamma oscillations is challenging [48], specifically at a young age [49]. This assumption does also correspond with the behavioral results in the present study, showing an increased accuracy for feature judgments during intentional encoding. Thus, participants might have focused their attention on perceptual details during the second, intentional encoding task, because they were aware that these would be relevant for subsequent retrieval [42]. In fact, when asked about the strategies they had used following each retrieval phase, all participants confirmed that they did not expect a memory test following incidental encoding and reported no particular encoding strategies. By contrast, 9 young adults, 6 older and 4 young children explicitly reported a strategy shift towards closely attending to item details during the intentional encoding phase.

In adults and older children parietal theta activity was higher when participants were instructed to explicitly memorize. By contrast, parietal theta did not differ between both phases in younger children. This may indicate changes in perceptual encoding around that age [50], and is in line with a recent fMRI study [36], suggesting that perceptual elaboration is particularly relevant for incidental encoding in young children. To summarize, when perceptual details are relevant for memory retrieval, the elaboration of perceptual details appears to be a critical mechanism that underlies intentional encoding of novel information across development. Perceptual binding processes may play a specific role in young children, also during incidental encoding.

Age-dependent decreases in alpha activity in frontal and posterior cortical networks presumably reflect developmental changes in semantic encoding processes [29]. These topographical properties of alpha suppression in young adults closely resemble subsequent memory effects during semantic encoding in adults [19, 20]. This finding supports the theoretical notion that mnemonic processing depends on increasingly abstract semantic representations throughout childhood [2]. In line with this view, posterior alpha suppression was prominent in students and less pronounced, but prolonged for children. The duration of this effect mirrored age differences in response times for the semantic (indoor-outdoor) encoding task. Thus, increased alpha suppression during encoding in adults possibly indicates a more efficient processing of new information, based on increasingly abstract representations.

Notably, alpha suppression may be associated with other cognitive processes beside memory encoding. More generally, alpha suppression has been associated with attention towards external stimuli [29]. It is conceivable that both semantic and attentional processing are closely linked during memory formation. Furthermore, because incidental encoding sessions were always conducted before the intentional phase to ensure that participants did not anticipate the memory test, it is possible that the order of the encoding tasks may also have contributed to the reduction of alpha suppression, due to lower attentional resources towards the end of the experiment. However, memory performance did not decline in the intentional compared to the incidental encoding condition. Thus, the present behavioral results do not suggest a strong reduction in attentional resources. Please note that, in the present study, successfully encoded items were not compared to those later forgotten, a comparison that would be interesting for future research.

### Similarities between memory encoding and retrieval

The present results complement previous findings demonstrating qualitative differences in memory processing of children and young adults as revealed by specific types of retrieval errors: By contrast to adults, who tend to falsely endorse semantically related lures in a Deese-Roedinger-McDermott paradigm, young children tend to endorse unrelated words that rhyme with presented items, suggesting that rote rehearsal is used for memory maintenance instead of semantic elaboration in this age group [33]. Likewise, previous studies investigating age differences during memory retrieval using event-related potentials (ERPs) found that children primarily rely on recollection-based memory retrieval processes, which support the retrieval of specific details, whereas young adults showed higher familiarity-based retrieval activity, closely related to conceptual processing, in addition to recollection-based memory retrieval [40, 43]. In line with previous findings [51], we found partially overlapping neuronal network activity during memory encoding and retrieval in the present paradigm [42, 43]. Thus, for young children, similar neuronal processes seem to be involved during memory formation and retrieval, which are gradually complemented by additional processes developing with increasing age [34].

### Incidental and intentional memory encoding processes

Analyzing neuronal oscillations during incidental learning allowed us to investigate cognitive processing of pictorial material when children and young adults were not aware of a subsequent memory test. Notably, as indicated by the behavioral results, i.e., no difference between age groups, the difficulty of the memory tasks was comparable between age groups. Thus, we were able to avoid differences in performance between age groups potentially confounding the associated neuronal activity. Despite similar memory performances, the underlying encoding processes differed between age groups: Children showed an age specific effect for perceptual encoding processes, while semantic encoding processes increased with age. Notably, during intentional encoding, participants performed the same orienting task, so both phases differed only in the fact that participants were now aware of the later retrieval task and of which stimulus features were relevant for the subsequent test. Intentional and incidental encoding processes led to very similar memory performance, suggesting that similar behavioral learning effects may be achieved via partially different routes. Notably, memory performance improved for task-relevant aspects, but not for overall memory performance. In particular the encoding of stimulus features increased, possibly due to the relevance for the particular task. Thus, as long as encoding operations encourage a deep processing of the material to be studied, such as semantic encoding, it is seems to be possible to encode high amounts of information without being aware of a later memory test [1].

## Conclusion

The present data suggest that intentional encoding relies on perceptual binding processes in both children and young adults, reflected in encoding-related increases in theta activity. Theta activity is assumed to reflect binding and ordering processes in cortical and medio-temporal networks, which constitute a key mechanism for episodic encoding. Young children showed an age specific effect, indicating that perceptual processes in parietal regions may be similarly relevant in incidental as in intentional encoding in this age group. Semantic processes during encoding increased with age, as reflected in pronounced alpha suppression in frontal and posterior cortical networks with age. The present pattern of results complement current empirical findings and theoretical accounts positing that increasingly abstract, semantic representations gradually complement perceptual binding operations throughout middle childhood.

## Supporting information

S1 File SPSS data. The data set contains all data included in the behavioral and EEG data analysis. Variable names correspond to the descriptions in the manuscript.
(SAV)
